# A prospective study of acute kidney injury in the intensive care unit: development and validation of a risk prediction model

**DOI:** 10.1186/s12967-019-2118-6

**Published:** 2019-11-05

**Authors:** Qi Wang, Yi Tang, Jiaojiao Zhou, Wei Qin

**Affiliations:** 1 Division of Nephrology, Department of Medicine, West China Hospital of Sichuan University, Chengdu, Sichuan China; 2 West China School of Medicine, Sichuan University, Chengdu, Sichuan China; 3 Division of Ultrasound, West China Hospital of Sichuan University, Chengdu, Sichuan China

**Keywords:** Risk prediction model, Acute kidney injury (AKI), Intensive Care Unit (ICU)

## Abstract

**Background:**

Acute kidney injury (AKI) has high morbidity and mortality in intensive care units (ICU). It can also lead to chronic kidney disease (CKD), more costs and longer hospital stay. Early identification of AKI is important.

**Methods:**

We conducted this monocenter prospective observational study at West China Hospital, Sichuan University, China. We recorded information of each patient in the ICU within 24 h after admission and updated every two days. Patients who reached the primary outcome were accepted into the AKI group. Of all patients, we randomly drew 70% as the development cohort and the remaining 30% as the validation cohort. Using binary logistic regression we got a risk prediction model of the development cohort. In the validation cohort, we validated its discrimination by the area under the receiver operator curve (AUROC) and calibration by a calibration curve.

**Results:**

There were 656 patients in the development cohorts and 280 in the validation cohort. Independent predictors of AKI in the risk prediction model including hypertension, chronic kidney disease, acute pancreatitis, cardiac failure, shock, pH ≤ 7.30, CK > 1000 U/L, hypoproteinemia, nephrotoxin exposure, and male. In the validation cohort, the AUROC is 0.783 (95% CI 0.730–0.836) and the calibration curve shows good calibration of this prediction model. The optimal cut-off value to distinguish high-risk and low-risk patients is 4.5 points (sensitivity is 78.4%, specificity is 73.2% and Youden’s index is 0.516).

**Conclusions:**

This risk prediction model can help to identify high-risk patients of AKI in ICU to prevent the development of AKI and treat it at the early stages.

*Trial registration* TCTR, TCTR20170531001. Registered 30 May 2017, http://www.clinicaltrials.in.th/index.php?tp=regtrials&menu=trialsearch&smenu=fulltext&task=search&task2=view1&id=2573

## Background

Acute kidney injury (AKI) is one of the most common complications in critically ill patients in intensive care units (ICU). The morbidity of AKI in ICUs can up to 50% according to some studies [1, 2]. In China, this problem also exists. The incidence of AKI in Chinese ICUs is approximately between 20 and 50% [3–6]. Many studies show that the occurrence of AKI can lead to worse prognosis, including high long-term mortality [7, 8] and the incidence of chronic kidney disease (CKD) [9, 10]. Furthermore, AKI might cause more costs and longer hospital stay [11–13].

Early identification of AKI is essential to critically ill patients because there’s no good therapy but renal replacement therapy (RRT) when it becomes severe. But RRT is a huge burden for critically ill patients.

There are a few studies developed risk prediction models to identify AKI, but each of these models has their own limitations [14–18]. We still need more studies from more centers to get a model which can predict the risk of AKI even more accurately. Then the clinicians can identify AKI early and precisely, and treat it before it becomes severe.

## Methods

This is a monocenter prospective observational study conducted at West China Hospital, Sichuan University, China. We had obtained informed consent from all participants.

### Data collection and classification

We recorded demographic information, medical history, physical examination, laboratory examination and therapeutic regimen of each patient in the intensive care unit within 24 h after admission of ICU. Laboratory examination results and therapeutic regimen were collected every two days.

The primary outcome variable was the development of AKI, which defined with a modified KDIGO serum creatinine (SCr) criterion (increasing 26.5 μmol/L within 48 h or becoming 1.5 times higher than the baseline within 7 days). The baseline of SCr was regarded as the SCr measured at ICU admission. The secondary outcome variables were death and receiving RRT.

**Fig. 1:**
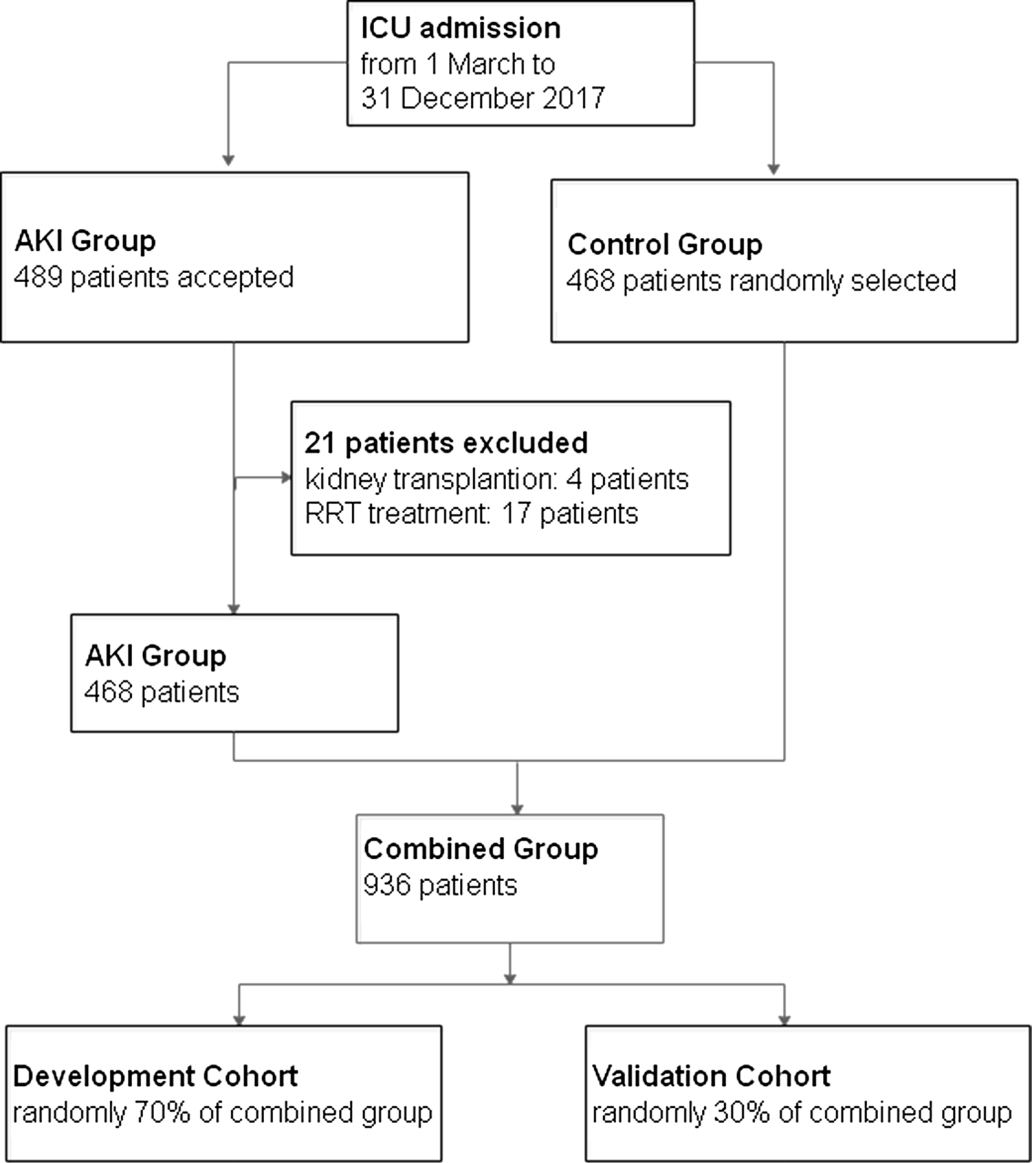
Participate flow chart of Study

Patients who reached the primary outcome and were age 14 years or older were accepted into the AKI group (489 patients) and were excluded if they used to have a kidney transplant or were receiving RRT. From 1 March 2017 to 31 December 2017, 468 patients were accepted into the AKI group. We randomly selected 468 patients who did not reach the primary outcome before discharging from the hospital as the control group. Considering that others’ researches [19–21] mostly divided the development and validation cohort patients as a ratio between 7:3 and 8:2, of the 936 patients from two groups, we randomly drew 70% as the development cohort and the remaining 30% as the validation cohort (Fig. 1).

### Risk factors

Basing on some other studies [22–27], we had chosen several candidate predictor variables including gender (male), age (> 70 years), hypertension, diabetes, chronic kidney disease, chronic liver disease, chronic pulmonary disease, coronary heart disease, cancer, acute pancreatitis, severe empyrosis, trauma, cardiac failure, respiratory failure, sepsis, shock, cardiopulmonary arrest, anemia (hemoglobin < 110 g/L), hypoproteinemia (serum albumin < 30 g/L), low blood pH (pH ≤ 7.30), high creatine kinase (CK > 1000 U/L), nephrotoxin exposure, major surgery and blood transfusion. Shock is defined as a life-threatening, generalized form of acute circulatory failure associated with inadequate oxygen utilization by the cells [28]. For ease of analysis and interpretation, we represented those risk factors as binary predictor variables.

### Statistical methods

Continuous variables were presented as the mean (SD) or median and interquartile range (IQR), depending on distribution, and were analyzed by unpaired *t* test. Categorical variables were presented as percentages and were analyzed using a Chi squared test.

Using binary logistic regression (forward: LR), we got independent predictors of the development of AKI and developed a risk prediction model of AKI. For ease of application, we turned the risk prediction model into a risk prediction score. We converted the coefficients into integers by converting the smallest coefficient to 1 and dividing other coefficients by the minimum coefficient and rounding the numbers to the nearest integers.

Because the performance in the development cohort may over-estimate the performance of other patients, we validated the risk prediction score in the validation cohort by estimating discrimination and calibration. Discrimination was estimated by the area under the receiver operator curve (AUROC) and calibration was evaluated using a calibration curve.

## Results

### Baseline characteristics

Table 1 shows the baseline characteristics of the development cohort and the validation cohort. A total of 656 patients were included in the development cohorts and 280 in the validation cohort. Patients’ mean ages are 58 (SD 18) years in the development cohort which has 68% (n = 445) males and 57 (SD 18) years in the validation cohort which has 69% (n = 193) males. Serum creatinine baseline measures were also similar between two cohorts (85 (IQR 62–131) umol/L in the development cohort, 80 (IQR 58–118) umol/L) in the validation cohort). In the development and validation cohorts, the prevalence of hypertension is 32% (n = 213) and 35% (n = 97), respectively. Among AKI patients, 32% (n = 106) patients in the development cohorts and 32% (n = 45) patients in the validation cohort received RRT. The hospital mortality of the patients who developed AKI is significantly higher than the patients without AKI in both cohorts (52% versus 13% in the development cohort and 49% versus 11% in the validation cohort). The development cohort and the validation cohort was compared using independent-sample t test in continuous variables (age and SCr baseline measures) and Chi square test in categorical variables (all risk factors). P-values are greater than 0.05 at all of the variables, which means they have no significant difference between the two cohorts.

**Table 1:** Baseline characteristics of patients in the development and validation cohorts

Variables	Development Cohort (n = 656)	Validation Cohort (n = 280)	P-value
Age, years, mean (SD)	58 (18)	57 (18)	0.445
Male, n (%)	445 (68)	193 (69)	0.742
SCr baseline measures, μmol/L, median (IQR)	85 (62–131)	80 (58–118)	0.264
Age > 70, n (%)	173 (26)	66 (24)	0.547
Hypertension, n (%)	213 (32)	97 (35)	0.518
Diabetes, n (%)	120 (18)	43 (15)	0.278
Chronic kidney disease, n (%)	50 (8)	24 (9)	0.622
Chronic liver disease, n (%)	73 (11)	35 (13)	0.547
Chronic pulmonary disease, n (%)	144 (22)	68 (24)	0.435
Coronary heart disease, n (%)	42 (6)	20 (7)	0.677
Cancer, n (%)	143 (22)	63 (23)	0.813
Acute pancreatitis, n (%)	75 (11)	36 (13)	0.537
Severe empyrosis, n (%)	8 (1)	4 (1)	0.795
Trauma, n (%)	162 (25)	56 (20)	0.120
Cardiac failure, n (%)	196 (30)	74 (26)	0.286
Respiratory failure, n (%)	310 (47)	122 (44)	0.300
Sepsis, n (%)	154 (23)	61 (22)	0.574
Shock, n (%)	273 (42)	122 (44)	0.579
Cardiopulmonary arrest, n (%)	52 (8)	15 (5)	0.163
Anemia, n (%)	530 (81)	218 (78)	0.305
Hypoproteinemia, n (%)	456 (70)	197 (70)	0.797
pH ≤ 7.30, n (%)	104 (16)	40 (14)	0.543
CK > 1000 U/L, n (%)	200 (30)	77 (28)	0.359
Nephrotoxin exposure, n (%)	131 (20)	69 (25)	0.110
Major surgery, n (%)	408 (62)	180 (64)	0.545
Blood transfusion, n (%)	330 (50)	133 (48)	0.432
Receiving RRT, n (%)	113 (17)	50 (18)	0.816
Mortality, n (%)	213 (32)	84 (30)	0.457

### Development of the risk prediction score

By univariate analysis, We selected 18 candidate predictors into a logistic regression analysis, with the development of AKI as the dependent variable. Those candidate predictors are gender (male), hypertension, diabetes, chronic kidney disease, chronic pulmonary disease, coronary heart disease, cancer, acute pancreatitis, trauma, cardiac failure, respiratory failure, sepsis, shock, cardiopulmonary arrest, hypoproteinemia, low blood pH (pH ≤ 7.30), high creatine kinase (CK > 1000 U/L), and nephrotoxin exposure.

Logistic regression analysis was conducted using forward Selection, and the outcome is showed in Table 2. Those 10 predictors entered the risk prediction model, including hypertension, chronic kidney disease, acute pancreatitis, cardiac failure, shock, pH ≤ 7.30, CK > 1000 U/L, hypoproteinemia, nephrotoxin exposure, and male.

**Table 2:** Predictors of AKI obtained by multivariate logistic regression in the development cohort

Variables	Coefficient	OR	95% CI for OR		P-value	Score
			Lower	Upper		
Hypertension	0.764	2.147	1.410	3.270	0.000	1
Chronic kidney disease	1.194	3.300	1.431	7.612	0.005	2
Acute pancreatitis	0.897	2.452	1.292	4.656	0.006	1
Cardiac failure	1.024	2.785	1.781	4.357	0.000	2
Shock	0.840	2.317	1.555	3.452	0.000	1
pH ≤ 7.30	1.067	2.906	1.652	5.111	0.000	2
CK > 1000U/L	1.272	3.568	2.327	5.471	0.000	2
Hypoproteinemia	1.240	3.457	2.218	5.388	0.000	2
Nephrotoxin exposure	0.752	2.122	1.294	3.481	0.003	1
Male	0.557	1.745	1.150	2.649	0.009	1

Then we turned the risk prediction model into a risk prediction score, showed in Table 2. The total score ranges from 0 to 15 points. Probability of ${\text{AKI}}\, = \, 1/\left[ { 1\, + \,{\text{EXP}}\left( { 2. 9 9 9{-}0. 5 5 7* {\text{total score}}} \right)} \right]$. The comparison table of total score and probability of AKI is showed in Table 3.

**Table 3:** The comparison table of total score and probability of AKI

Score	Probability of AKI	Score	Probability of AKI
0	0.047	8	0.811
1	0.080	9	0.882
2	0.132	10	0.929
3	0.209	11	0.958
4	0.316	12	0.976
5	0.447	13	0.986
6	0.585	14	0.992
7	0.711	15	0.995

**Fig. 2:**
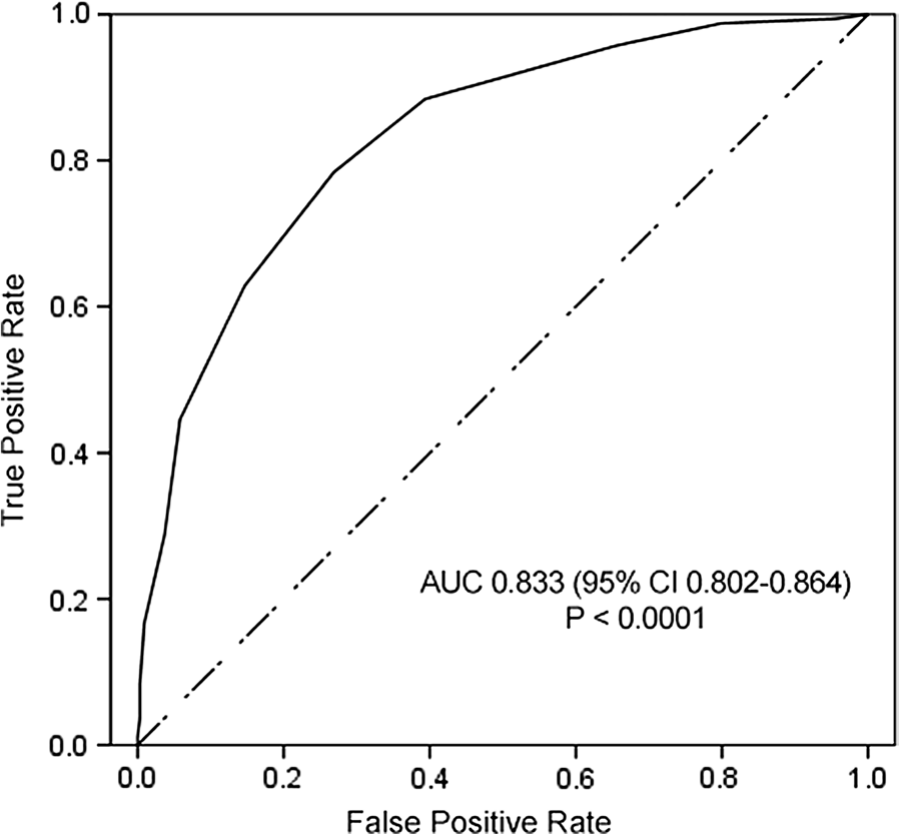
Area under the receiver operating characteristic of the development cohort

Hosmer–Lemeshow goodness-of-fit test’s P-value is 0.511. The area under the receiver operating characteristic of the development cohort is 0.833 (95% CI 0.802–0.864) (Fig. 2). Value is greater than 0.8 which indicates excellent predictive performance.

### Model validation

**Fig. 3:**
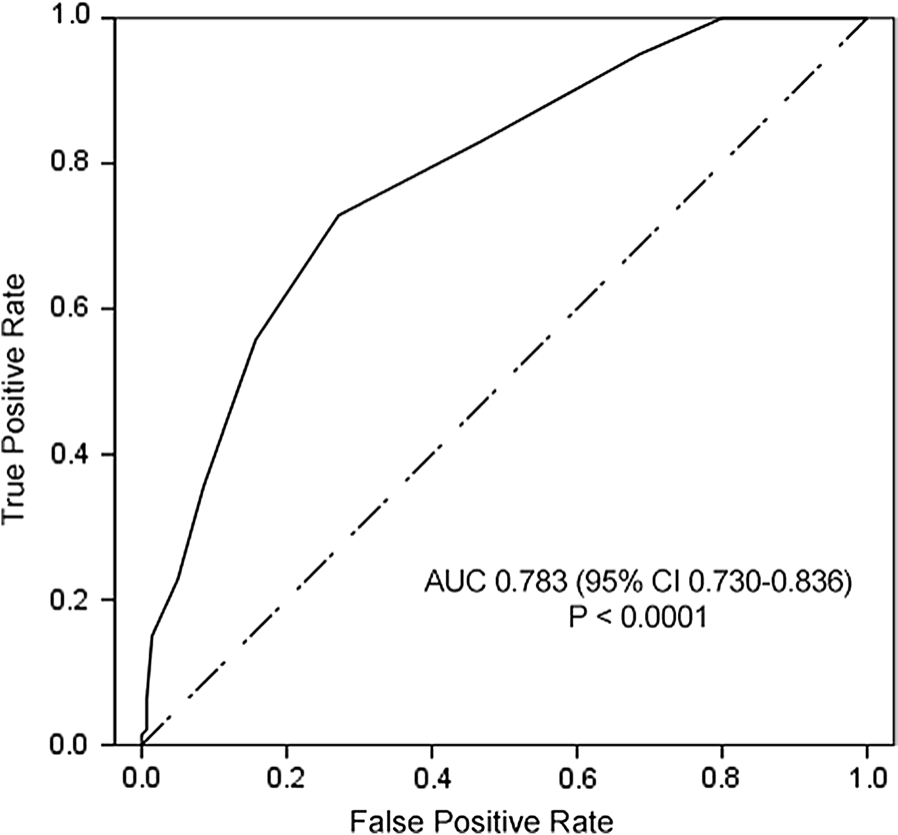
Area under the receiver operating characteristic of the validation cohort

**Fig. 4:**
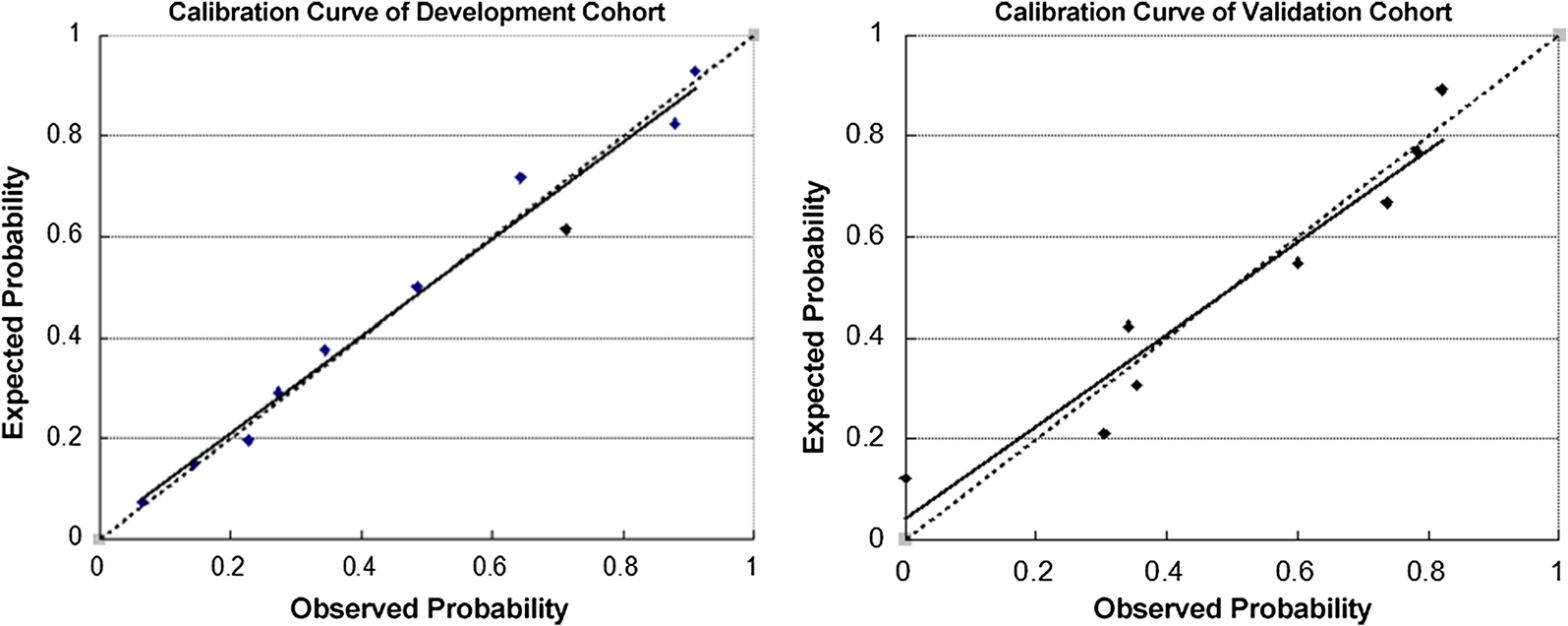
Calibration curve of development and validation cohorts

The area under the receiver operating characteristic of the validation cohort is 0.783 (95% CI 0.730–0.836) (Fig. 3). Value is greater than 0.7 which indicates good predictive performance. Calibration curves of development and validation cohorts (Fig. 4) show good calibration of this prediction score.

The optimal cut-off value to distinguish high-risk and low-risk patients is 4.5 points (sensitivity is 78.4%, specificity is 73.2% and Youden’s index is 0.516). This means that if a patient with a score of greater or equal to 5, this patient might have a high risk of developing AKI.

## Discussion

Acute kidney injury has high morbidity and mortality, especially in ICUs. For the purpose of early identification of AKI, researching on the risk prediction model of AKI is necessary. Existing models have many flaws. This model made up for some defects and proposed a few new risk prediction factors.

Clinically, if all the risk factors are identified and quantified to use to profile risk, 20%–30% AKI can be predicted and avoided [29]. After admission to a hospital, early prediction of AKI can bring high opportunity to prevent patients from developing AKI. There have been a few prediction models for AKI risk using in ICUs, but there are still many researchers trying to develop risk prediction models because we still need more sensitive and more accurate models to apply to the clinic.

We used logistic regression to develop a model. Of all the candidate variables, only a few turned out to predict AKI (hypertension, chronic kidney disease, acute pancreatitis, cardiac failure, shock, pH ≤ 7.30, CK > 1000 U/L, hypoproteinemia, nephrotoxin exposure, and male). In our analysis, some variables had high significance in univariate analysis (age > 70 years, diabetes, chronic liver disease, coronary heart disease, cancer, trauma, respiratory failure, sepsis, and major surgery), but were not selected into the final model. This means there may be some false associations or indirect associations between these variables and independent predictors. These variables turned out not independent predictors in this model. However, there are previous reports showing their relationship with the development of AKI. We still need more research about these variables.

Using risk ranges, a total score of 0–4 points was associated with a low risk of AKI. Patients with a total score of 5–15 have a high risk to develop AKI. However, this is not absolute. In our study, 22% of low-risk patients also developed AKI, which indicates that this risk prediction score is not perfect yet. In the following work, more efforts should be pay to include more variables and perform more accurate verification to perfect it.

There are several strengths of our study. Firstly, this is a prospective observational study. We collected data prospectively to ensure the information detailed and reliable and to reduce the impact of bias. Secondly, we included a lot of candidate risk factors in our research. The risk factors we have included are very comprehensive. Most of those which reported in previous researches have been included. All of them are medical history data and clinical examinations easily to get, which are convenient for clinicians to apply. With only 10 variables in the risk prediction score, it is simple to calculate. Thirdly, two variables (acute pancreatitis and CK > 1000 U/L) in our risk prediction score have not been included in other models before. There is a study [30] which reported the relationship between acute pancreatitis and AKI but there is no study included acute pancreatitis into the model. The incidence of acute pancreatitis in China is relatively high and the main causes of acute pancreatitis are different from those in Europe and America. Our study raises the possibility that acute pancreatitis in China is highly correlated with the occurrence of AKI. There is also a study [14] suggesting the correlation between rhabdomyolysis and AKI but no study include incorporated rhabdomyolysis into the model. We considered that it might be that rhabdomyolysis’ diagnostic criteria led to this result. We relaxed the standard of CK to greater than 1000 and found it highly correlated with the occurrence of AKI. Finally, the risk factors we included are similar to other studies, but which incorporated into the model were different. That may indicate that the risk factors for AKI differ depending on the regions and races. It provides a direction for future researchs.

However, our study also has several limitations. The most important one is that this is a monocenter study. We did not conduct externally verification and only verified internally. This may cause some problems with the extrapolation of our model and the results may not be widely generalizable in other regions and races. Secondly, recent studies emphasized the importance of AKI biomarkers to predict AKI [31, 32]. However, for ease of application, we did not include biomarkers because they are not yet widely used clinically. Thirdly, KDIGO criterion diagnoses AKI with both urine volume and serum creatinine. Nevertheless, we used serum creatinine only on account of the inaccuracy of urine volume data. Finally, In order to facilitate statistics, we represent most of the variables as binary variables, and the severity of each variable is not taken into account.

Prediction of AKI still has high importance because it is associated with high mortality and high morbidity. Our future studies will focus on improving our model by expanding the sample size and performing external validation. We may bring in some refined biomarkers as predictors.

There is no single intervention can improve the outcome of AKI patients, so a risk prediction model would most likely to be used as a measure to help those patients. On this road, we should all work harder because there is still a long way to go.

## Conclusion

We developed a risk prediction model which can help to identify high-risk patients of AKI in ICU to prevent the development of AKI and treat it at the early stages. The variables are easy to get and the score is liable to calculate. However, it also has several limitations. We will conduct further research to improve this model.

## Data Availability

The datasets generated and/or analysed during the current study are not publicly available due to privacy policy but are available from the corresponding author on reasonable request.

## Abbreviations

AKI: acute kidney injury
ICU: intensive care units
CKD: chronic kidney disease
RRT: renal replacement therapy
KDIGO: kidney disease: improving global outcomes
SCr: serum creatinine
CK: creatine kinase
AUROC: the area under the receiver operator curve

## References

[1] Doi Kent (2016). Role of kidney injury in sepsis. J Intensive Care.

[2] Darwin T, Fernanda V, Daniela A (2017). Epidemiology of acute kidney injury and chronic kidney disease in the intensive care unit. Revista Brasileira de Terapia Intensiva..

[3] Tang X, Chen D, Yu S (2017). Acute kidney injury burden in different clinical units: data from nationwide survey in China. PLoS ONE.

[4] Wang F, Pan W, Wang H (2014). The impacts of thyroid function on the diagnostic accuracy of Cystatin C to detect acute kidney injury in ICU patients: a prospective, observational study. Crit care (London, England).

[5] Zhou J, Yang L, Zhang K (2012). Risk factors for the prognosis of acute kidney injury under the acute kidney injury network definition: a retrospective, multicenter study in critically ill patients. Nephrology.

[6] Wang Na, Jiang Li (2015). Fluid balance and mortality in critically ill patients with acute kidney injury: a multicenter prospective epidemiological study. Crit Care.

[7] Daley J (1998). Independent association between acute renal failure and mortality. Am J Med..

[8] Hoste EA, Kellum JA (2004). Acute renal failure in the critically ill: impact on morbidity and mortality. Contrib Nephrol..

[9] Waikar SS, Liu KD, Chertow GM (2008). Diagnosis, Epidemiology and Outcomes of acute kidney injury. Clin J Am Soc Nephrol.

[10] Chawla LS, Amdur RL, Amodeo S (2011). The severity of acute kidney injury predicts profession to chronic kidney disease. Kidney Int.

[11] Dasta JF, Kanegill SL, Durtschi AJ (2008). Costs and outcomes of acute kidney injury (AKI) following cardiac surgery. Nephrol Dial Transplant..

[12] Chertow GM (2005). Acute kidney injury, mortality, length of stay, and costs in hospitalized patients. J Am Soc Nephrol.

[13] Brandt MM, Falvo AJ, Rubinfeld IS (2007). Renal dysfunction in trauma: even a little costs a lot. J Trauma: Inj Infect Crit Care.

[14] Malhotra R, Kashani KB, Macedo E (2017). A risk prediction score for acute kidney injury in the intensive care unit. Nephrol Dial Transplant.

[15] Palomba H, De Castro I, Neto ALC (2007). Acute kidney injury prediction following elective cardiac surgery: AKICS score. Kidney Int.

[16] Thakar CV, Arrigain S, Worley S (2005). A clinical score to predict acute renal failure after cardiac surgery. J Am Soc Nephrol.

[17] Kiers HD, Van den BM, Schoenmakers MCJ (2013). Comparison and clinical suitability of eight prediction models for cardiac surgery-related acute kidney injury. Nephrol Dial Transplant..

[18] Mehran R, Aymong ED, Nikolsky E (2004). A simple risk score for prediction of contrast-induced nephropathy after percutaneous coronary intervention: development and initial validation. J Am Coll Cardiol..

[19] Lei Z, Li J, Wu D (2016). Nomogram for preoperative estimation of microvascular invasion risk in hepatitis B virus-related hepatocellular carcinoma within the milan criteria. JAMA Surg.

[20] Hippisleycox J, Coupland C (2015). Development and validation of risk prediction algorithms to estimate future risk of common cancers in men and women: prospective cohort study. BMJ Open.

[21] Malhotra R, Kashani KB, Macedo E (2017). A risk prediction score for acute kidney injury in the intensive care unit. Nephrol Dial Transplant..

[22] Grams ME, Sang Y, Ballew SH (2015). A Meta-analysis of the association of estimated gfr, albuminuria, age, race, and sex with acute kidney injury. Am J Kidney Dis.

[23] Leblanc M, Kellum JA, Gibney RTN (2005). Risk factors for acute renal failure: inherent and modifiable risks. Curr Opin Crit Care.

[24] Kuiper JW, Groeneveld ABJ, Slutsky AS (2005). Mechanical ventilation and acute renal failure. Crit Care Med.

[25] Interrelationship of preoperative anemia (2015). intraoperative anemia, and red blood cell transfusion as potentially modifiable risk factors for acute kidney injury in cardiac surgery: a historical multicentre cohort study. Can J Anesthesia.

[26] Varrier M, Ostermann M (2014). Novel risk factors for acute kidney injury. Curr Opin Nephrol Hypertens.

[27] Nisula S, Kaukonen KM, Vaara ST (2013). Incidence, risk factors and 90-day mortality of patients with acute kidney injury in Finnish intensive care units: the FINNAKI study. Intensive Care Med..

[28] Cecconi M, Debacker D, Antonelli M (2014). Consensus on circulatory shock and hemodynamic monitoring. Task force of the European Society of Intensive Care Medicine. Intensive Care Med..

[29] Kate RJ, Perez RM, Mazumdar D (2016). Prediction and detection models for acute kidney injury in hospitalized older adults. BMC Med Inform Decis Mak.

[30] Petejova N, Martinek A (2013). Acute kidney injury following acute pancreatitis: a review. Biomed Pap..

[31] Lieske JC, Chawla L, Kashani K (2014). Biomarkers for acute kidney injury: where are we today? where should we go?. Clin Chem.

[32] Murray PT, Mehta RL, Shaw A (2014). Potential use of biomarkers in acute kidney injury: report and summary of recommendations from the 10th acute dialysis quality initiative consensus conference. Kidney Int.

